# The India Hypertension Control Initiative–early outcomes in 26 districts across five states of India, 2018–2020

**DOI:** 10.1038/s41371-022-00742-5

**Published:** 2022-08-09

**Authors:** Prabhdeep Kaur, Abhishek Kunwar, Meenakshi Sharma, Kiran Durgad, Sudhir Gupta, India Hypertension Control Initiative collaboration, Sampada D. Bangar, Vishwajit Bharadwaj, Rupali Bharadwaj, Sailaja Bitragunta, Sreedhar Chintala, Tapas K. Chakma, Deenadayalan Chandran, Tejpalsinh A. Chavan, Sunil Dar, Bidisha Das, R. S. Dhaliwal, Sandeep Singh Gill, Bipin Gopal, A. Branch Immanuel, Tanu Jain, Padmaja Jogewar, Chakshu Joshi, Abhishek Khanna, Suhas N. Khedkar, Ashish Krishna, Navneet Kumar, Vijay Kumar, M. Madhavi, Parasuraman Ganeshkumar, Anupam Khungar Pathni, Satyendra N. Ponna, Yannick P. Puthussery, Mohamed E. Rafique, Sivasubramanian Ramakrishnan, Sravan K. Reddy, Gopinath T. Sambandam, Swagata K. Sahoo, Ashish Saxsena, Bhawna Sharma, Suyesh Shrivastava, Azhagendran Sivalingam, Shweta Singh, Gurinder Bir Singh, Sunny Swarnkar, Jatin Thakkar, Fikru T. Tullu, Vettrichelvan Venkatasamy, Mohammed Wassey, Amol B. Wankhede, Balram Bhargava

**Affiliations:** 1 Division of Noncommunicable Diseases, ICMR-National Institute of Epidemiology, Chennai, India; 2 Dept of Noncommunicable Diseases, WHO Country Office for India, New Delhi, India; 3 Indian Council of Medical Research (ICMR), New Delhi, India; 4 Directorate General of Health Services, Ministry and Health, and Family Welfare, New Delhi, India; 5 ICMR-National AIDS Research Institute, Pune, India; 6 IHCI Project, District NCD Cell, Bhandara (Maharashtra), WHO-India, Maharashtra, India; 7 IHCI Project, District NCD Cell, Chhindwara (Madhya Pradesh), WHO-India, Madhya Pradesh, India; 8 IHCI Project, District NCD Cell, Karimnagar (Telangana), WHO-India, Telangana, India; 9 ICMR-National Institute of Research in Tribal Health, Jabalpur, India; 10 IHCI Project, District NCD Cell, Kannur (Kerala), WHO-India, Kerala, India; 11 IHCI Project, District NCD Cell, Sindudurg (Maharashtra), WHO-India, Maharashtra, India; 12 IHCI Project, District NCD Cell, Hoshiarpur (Punjab), WHO-India, Punjab, India; 13 IHCI Project, District NCD Cell, Bhatinda (Punjab), WHO-India, Punjab, India; 14 State NCD Cell, Department of Health and Family Welfare, Government of Punjab, Chandigarh, India; 15 State NCD Cell, Directorate of Health Services, Government of Kerala, Thiruvananthapuram, India; 16 IHCI Project, District NCD Cell, Thiruvananthapuram (Kerala), WHO-India, Kerala, India; 17 State NCD Cell, Directorate of Health Services, Government of Maharashtra, Mumbai, India; 18 IHCI Project, District NCD Cell, Ratlam (Madhya Pradesh), WHO-India, Madhya Pradesh, India; 19 NPCDCS Cell, DGHS, New Delhi, WHO- India, New Delhi, India; 20 IHCI Project, District NCD Cell, Satara (Maharashtra), WHO-India, Maharashtra, India; 21 Resolve to Save Lives, New Delhi, India; 22 IHCI Project, State NCD Cell, Chandigarh (Punjab), WHO-India, Punjab, India; 23 IHCI Project, District NCD Cell, Gurdaspur (Punjab), WHO-India, Punjab, India; 24 State NCD Cell, Department of Health, Medical and Family Welfare, Government of Telangana, Hyderabad, India; 25 IHCI Project, District NCD Cell, Warangal (Telangana), WHO-India, Telangana, India; 26 IHCI Project, District NCD Cell, Thrissur (Kerala), WHO-India, Kerala, India; 27 IHCI Project, District NCD Cell, Wayanad (Kerala), WHO-India, Kerala, India; 28 All India Institute of Medical Sciences, New Delhi, India; 29 IHCI Project, State NCD Cell, Hyderabad (Telangana), WHO-India, Telangana, India; 30 IHCI Project, District NCD Cell, Kannur (Kerala), WHO-India, Kerala, India; 31 State NCD Cell, Directorate of Health Services, Government of Madhya Pradesh, Bhopal, India; 32 IHCI Project, State NCD Cell, Bhopal (Madhya Pradesh), WHO-India, Madhya Pradesh, India; 33 IHCI Project, District NCD Cell, Mahabubnagar (Telangana), WHO-India, Telangana, India; 34 IHCI Project, State NCD Cell, Mumbai (Maharashtra), WHO-India, Maharashtra, India

**Keywords:** Medical research, Hypertension

## Abstract

Hypertension is the leading single preventable risk factor for cardiovascular disease. The India Hypertension Control Initiative (IHCI) project was designed to improve hypertension control in public sector clinics. The project was launched in 2018–2019 in 26 districts across five states: Punjab (5), Madhya Pradesh (3), Kerala (4), Maharashtra (4), and Telangana (10), with five core strategies: standard treatment protocol, reliable supply of free antihypertensive drugs, team-based care, patient-centered care, and an information system to track individual patient treatment and blood pressure control. All states implemented simple treatment protocols with three drugs: a long-acting dihydropyridine calcium channel blocker (amlodipine), angiotensin receptor blocker (telmisartan), and thiazide or a thiazide-like diuretic (hydrochlorothiazide or chlorthalidone). Medication supplies were adequate to support at least one month of treatment. Overall, 570,365 hypertensives were enrolled in 2018–2019; 11% did not have follow-up visits in the most recent 12 months. Clinic-level blood pressure control averaged 43% (range 22–79%) by Jan-March, 2020. The proportion of the estimated people with hypertension who had it controlled and documented in public clinics increased three-fold, albeit from very low levels (1.4–5.0%). The IHCI demonstrated the feasibility of implementing protocol-based hypertension treatment and control supported by a reliable drug supply and accurate information systems at scale in Indian primary health care facilities. Lessons from the IHCI’s initial phase will inform plans to improve screening in health care facilities, increase retention in care, and ensure a sustained supply of drugs as part of a nationwide hypertension control program.

## Introduction

Cardiovascular diseases (CVD) were among the five leading causes of total disease burden in India in 2016. [1] High blood pressure is the leading single risk factor for CVD in India. [1] Blood pressure control using low-cost, generic antihypertensive drugs is considered by the World Health Organisation (WHO) to be one of the “best buys” for NCD control. Although hypertension can be treated in primary health care facilities with low-cost, generic medications, achieving hypertension control at the community level is difficult. Some high-income countries have improved population hypertension control through community and clinic-based interventions. For example, Canada improved population-wide hypertension control from 13.2% in 1992 to 64.6% in 2009. [2] Among middle-income countries, Thailand increased population hypertension control from 8.6% in 2004 to 30% in 2014. [3]

WHO developed a Global Monitoring Framework to assess progress in controlling noncommunicable diseases in 2013. The WHO framework targets include a 25% relative reduction in overall mortality from cardiovascular diseases and a 25% relative reduction in the prevalence of raised blood pressure by 2025. [4] The Government of India (GOI) adopted the Global Monitoring Framework and developed a national action plan to prevent and control noncommunicable diseases (NCDs) to achieve the targets. The India Hypertension Control Initiative (IHCI) was initiated as a collaborative project of the Ministry of Health and Family Welfare (MoHFW), State Governments, the Indian Council of Medical Research (ICMR), and the World Health Organization (WHO), along with a technical partnership with the global non-profit organization Resolve to Save Lives. The overall aim of the IHCI is to improve hypertension management and increase hypertension control from an estimated 10% to at least 30% among all people with hypertension over five years.

The IHCI works closely with the Indian National Health Mission (NHM) and its chronic disease program, the National Program for Prevention and Control of Cancer, Diabetes, CVD, and Stroke (NPCDCS), to strengthen the appropriate management of hypertension and the continuum of hypertension care. [5] Phase I of the IHCI project was launched in November 2017 and implemented in 26 districts in five states in the first year. This article documents the design of IHCI Phase I and its progress and challenges to date, including the implementation of program strategies and estimates of blood pressure control at the public sector clinics and among all people estimated to have hypertension in the IHCI districts.

## Methods

### Project sites and health facilities

The project was implemented in 26 districts across five Indian states: Punjab (5), Madhya Pradesh (3), Kerala (4), Maharashtra (4), and Telangana (10). Adult patients visiting public sector health facilities were registered after screening and diagnosis during routine health care visits and thereafter initiated treatment.

All levels of health facilities were included in the intervention. The district and sub-district hospitals cater to the entire district for secondary care services, and Community Health Centres (CHC) each cater to approximately 100,000 people. Primary Health Centres (PHC) cover 30,000 population. PHCs have 5–6 sub-centres where field-level health workers provide services (primarily maternal and child health) to a population of approximately 5000. In recent years, the Government of India and state governments have begun upgrading sub-centres to Health and Wellness Centres to improve access to primary care services. [6] Health and Wellness Centres have a specially trained nurse (Community Health Officer) and provide primary care services, including, in IHCI districts, outpatient care for hypertension.

### The IHCI intervention

#### Core strategies

We adapted the WHO HEARTS technical package for hypertension control programs to suit the local needs. [7] The project aimed to implement five strategies

1. Protocols: Use of standard drug- and dose-specific protocols for hypertension management agreed upon by various stakeholders during consensus conferences at the state level and consistent with national and global policies.
2. Drugs: Ensure availability of drugs included in the state protocol in all facilities with at least three months’ stock of medications at all times.
3. Team-based care: Capacity building and involvement of all health staff levels in delivering hypertension management, including nurses in health facilities and health workers/ASHA (Accredited Social Health Activists) at the community level.
4. Patient-centered services: High-quality service delivery in all health facilities using various approaches such as the provision of decentralized hypertension services down to the most peripheral, community-level facilities, documentation of visits in the treatment card or digital app, BP monitoring, minimum 30-day free drug refills, counseling, and free medications.
5. Monitoring systems: Standard indicators and documentation mechanisms ensure standardized data collection for the key monitoring indicators, primarily the number of patients with controlled blood pressure and blood pressure control rates.

#### Screening, treatment, and follow-up

Strategies were implemented within the existing healthcare system involving various levels of healthcare worker staff. One nurse is routinely posted under the NPCDCS or assigned from existing state-level health staff. Under the IHCI project, NCD corners/stations were set up in most facilities to promote opportunistic hypertension screening of patients attending health facilities for non-emergency reasons and streamline patient flow. Additionally, responsible health care staff (usually staff nurses) were trained in appropriate BP measurement methods, and BP measurement checklist posters were provided to all clinics in the local languages. [8] Nurses performed opportunistic screening, blood pressure monitoring during follow-up visits, registered patients using the treatment card or app, counseling for lifestyle modification, archived and retrieved patient treatment cards during follow-up visits. Doctors prescribed the drugs as per the protocol. The Health Wellness Centres are staffed by a mid-level health provider (Community Health Officer), a specially trained nurse, or other practitioner who provides NCD services, including screening for hypertension and diabetes, blood pressure monitoring, and distributing drugs for follow-up patients initiated treatment in higher-level facilities.

In the classroom-based training program, the project team trained more than 80% of medical officers, nurses, and other paramedical staff at health facilities using easy-to-follow job aids and protocol posters for ready reference in health care facilities. [8]

#### Information system

The IHCI project designed paper-based and digital app-based monitoring systems to improve documentation of critical indicators and feedback to stakeholders, particularly the level of blood pressure control, based on all patients registered with hypertension. The paper-based information systems for hypertension care were modeled after the WHO HEARTS technical package to monitor the patient cohort for treatment outcomes. [7] In states and districts using the paper-based system, a treatment card, and a register were maintained in the health facilities. In the last quarter of 2018, Punjab and Maharashtra introduced the Simple app, a hand-held mobile device application designed to quickly and accurately register patients, record blood pressures over time, and record medication regimens. [9]

#### Supportive supervision

Cardiovascular Health Officers (CVHO) and Senior Treatment Supervisors (STS), recruited under the project, facilitated implementation, conducted 10–15 supervisory visits per month each, using a supervisory checklist, and provided timely feedback to stakeholders. They submitted monthly reports to State and District health officials that summarised program implementation progress, key achievements and challenges.

### Data collection

For this report, we extracted the information regarding progress and challenges from monthly reports and reports of field supervision visits. Data regarding the number of patient registrations, treatment progress, and drug stocks at the state, district, and health facility level were collected. Aggregated data regarding follow-up visits and blood pressure control were collected every quarter from patient cards maintained in health facilities. Monthly aggregate data were also collected from the Simple app-based information system in Maharashtra and Punjab.

### Data analysis and operational definitions

We analyzed monthly cumulative patient enrolment data, district population sizes, and the prior population-based survey hypertension prevalence estimates used to estimate the proportion of estimated people living with hypertension registered in the program. Blood pressure at the most recent visit during the first quarter of 2020 was extracted from patient cards in three states and the “Simple app” database for Punjab and Maharashtra. Operational definitions of key indicators included in the analysis are described here.

### Facility level indicators

- Registrations: Number of patients with hypertension enrolled under the program in 2018–19 after being diagnosed by the Medical Officer. Registrations include patients already taking antihypertensive medications and newly diagnosed patients during screening.
- Patients under care: Patients with hypertension who had at least one visit (new or follow-up) to a health care facility over 12 months between 1st April 2019 and 31st March 2020.
- Drugs availability inpatient days: Number of days the drug stock will last given the number of patients under care in a facility/district and based on the estimates of proportions at each step in the treatment protocol.
- Blood pressure under control: Systolic BP < 140 mmHg and diastolic BP < 90 mmHg during a most recent visit in the quarter 1st January 2020–31st March 2020, among all patients under care.
- Blood pressure not under control: Systolic BP > = 140 mmHg or diastolic BP > = 90 mmHg during a most recent visit in the quarter 1st January 2020–31st March 2020, among all patients under care.
- Lost to follow up: Registered patients with hypertension who did not have a follow-up visit over 12 months between 1st April 2019 and 31st March 2020.
- Missed visit: Registered patient with hypertension who had no recorded visit in one reporting quarter, namely 1st January 2020–31st March 2020.

### Community-level indicator

- Community-level blood pressure control: Proportion of estimated population with hypertension in a given geographical area (district or state), with controlled blood pressure based on data in public sector facilities (measured annually). The numerator was the number of patients with controlled blood pressure in all the health facilities from 1st January 2020 to 31st March 2020. The denominator was the estimated number of patients in the district. The number was computed using the projected population based on the Census of India and prevalence estimate from community surveys. [10]

### Human subjects protection

The Institutional Ethics Committee of ICMR-National Institute of Epidemiology approved the project protocol. Patient-level data were maintained at the health facility and accessed only by care providers. Cumulative facility-level data were collected, de-identified, and analyzed in aggregate for key indicators at the district, state, and national levels.

## Results

### Drug- and dose-specific hypertension treatment protocols

All five states developed and implemented drug- and dose-specific hypertension treatment protocols (Table 1). [11] To develop the treatment protocols, NCD program managers conducted consensus workshops in Phase 1 states. The workshops included health officials from the state health departments, clinicians from the District hospitals, specialists from medical colleges, and external invited experts. All protocols included specific doses of three drugs: the calcium channel blocker amlodipine, the angiotensin receptor blocker telmisartan, and the diuretic chlorthalidone (or hydrochlorothiazide). The six steps in protocol guided the progressive addition of dose/drugs needed to control BP and facilitated a realistic estimation of drug supply requirements and accelerated procurement.

**Table 1:** Description of progress and challenges related to core implementation strategies of India Hypertension Control Initiative, India, 2018–2020.

	Description	Progress	Challenges
Drug/dosage specific protocol	Three drugs included in the protocols - Amlodipine (5 mg and 10 mg), Telmisartan (40 mg and 80 mg) and a diuretic (either Hydrochlorothiazide or Chlorthalidone).	Protocol finalized in the State consensus meetings. State experts/doctors in the primary health care facilities found drug and dose specific protocols were useful and simple	There was reluctance among doctors to add another drug or escalate the dose which was more evident in few districts.
Availability of adequate drugs and blood pressure monitors	Drug requirement estimated using the forecasting tool and procurement done through state level procurement agencies.	Drugs procured as per protocols and adequate stock for one month refills in all districts.	Three month buffer stock not availble in all health facilities. Refills for more than one month could not be operationalised.
	Validated professional digital BP monitors provided in the project districts.	Acceptance of digital BP monitors improved among health care providers.	Few doctors not convinced about the accuracy of digital BP monitors and procurement is challenging due to cost and administrative reasons.
Team based care and task sharing	Team based approach involving doctors and nurses in the clinic. Opportunistic screening, BP measurement and documentation in a specially set NCD corner in the health facilities	The tasks distributed between doctor, nurse and pharmacist at clinics. Patient flow streamlined in many facilities to ensure all patients have BP checked and card entry made by nurse. Pharmacist ensures BP measurement done before dispensing drugs.	Lack of adequate staff to check blood pressure of all patients for opportunistic screening and patients directly visiting the doctor for prescription in some of the health facilities.
Decentralised patient centered care	Blood presssure measurement and refills for at least 30 days at all health fcailities including Health Wellness centers or sub centers closer to home.	Health wellness centers/ Sub centers operationalised drug refillls in three states - Punjab, Madhya Pradesh and Maharashtra	Extended refills for more than 30 days not yet universally approved nor logistically feasible.
Information systems, monitoring and supervision	A paper based and an android based “Simple” app used for enrollment and follow up.	Paper based system (patient card and a line list register) adopted by Madhya Pradesh, Telangana and Kerala. Simple app used in Punjab and Maharashtra.	Card retrieval difficult in crowded facilities and retrieval of data from cards time consuming for quarterly and annual reports.
	Supervision and monitoring planned with dedicated staff who used a standardised supervision checklist while visiting health facilities.	20 CVHO and 38 STS (each STS visits at least 15 health facilities) visit all health facilities for supportive supervision and provide feedback to the doctors and prohram amangers	Low priority for NCD program and lack of trained district NCD nodal officers for program monitoring

### Ensuring availability of antihypertensive drugs and validated BP monitors

**Fig. 1:**
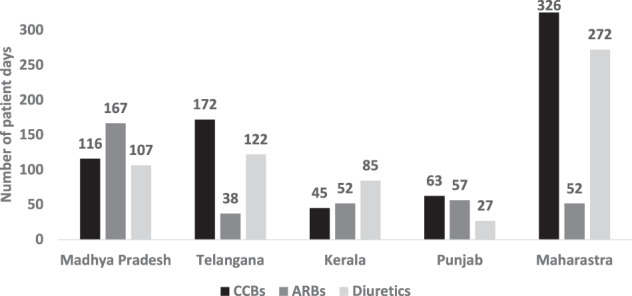
Availability of antihypertensive drugs in the IHCI project sites, March 2020. This chart describes the availability of three types of antihypertensive drugs in patient days (≥90 days is optimal) in five Indian states.

Soon after the program launch, shortages and stockouts of the protocol drugs at service delivery points emerged as significant challenges. In addition to inherent difficulties with public procurement, a lack of experience in handling large-scale public health programs requiring a lifelong supply of medications increased the difficulty in planning and procurement for the state procurement agencies. IHCI’s efforts to ensure accurate supply forecasting, budget allocations, timely procurement, and distribution of drugs gradually improved the availability of medicines in all districts (Table 1). At the district level and in the health facilities, all three protocol drugs were available for more than 30 patient days, ensuring one-month refills continuously for each patient (Fig. 1).

The project initially provided professional, independently-validated digital, automated digital blood pressure monitors meeting WHO-recommended specifications to most facilities to demonstrate the value of good quality measurement devices and encourage task sharing. [12] Based on positive feedback from using these good-quality BP monitors, several states have now initiated steps to procure additional professional validated devices using state resources.

### Team-based and patient-centric hypertension care

The project was implemented in 1417 facilities by December 2020, including 63 district/sub-district hospitals, 170 Community Health Centres (CHC), 864 Primary Health Centres (PHC) and 320 Health Wellness Centres (HWC). All five states implemented team-based care involving nurses, doctors and pharmacists to manage hypertension (Table 1). Lack of dedicated nurses and vacancies of doctor posts, especially in Punjab and Madhya Pradesh, limited the increase in patient registrations and follow-up in several health facilities. Opportunistic hypertension screening was implemented with limited success except in Telangana and two districts of Maharashtra, where dedicated NCD nurses at the PHCs enabled daily screening. Many of the patients attending busy health care facilities, particularly hospitals, did not have blood pressure taken, and, when taken, many with elevated readings were not started on treatment. During supportive supervision visits, the project team advised ways to streamline the flow of patients through facilities so that patients first visited the nurse, who measured the blood pressure and updated the medical record before the patient visited the doctor for evaluation and a prescription.

All five states implemented drug dispensing for 30-days across all types of health facilities (Table 1). Patient follow-up was decentralized to 320 HWC based in the patient’s community. In Health Wellness Centres, the Community Health Officer (CHO) measured blood pressure and provided refills.

### Information systems and key indicators

Data was collected using paper-based cards in Kerala, Madhya Pradesh, and Telangana. The Simple app, an android based app, was introduced in Punjab and Maharashtra. The transition to this mobile phone-based app reduced paperwork, improved documentation of blood pressures, facilitated a more rapid generation of program monitoring reports, and enabled quick feedback on program performance indicators to facilities and districts via summary dashboards. Using Simple, a health worker completes a new patient registration in <60 s and documents a follow-up visit in <20 s. Existing patient data can be retrieved in <5 s by scanning BP passport cards with unique QR codes and carried by the patient. High-risk patients are automatically prioritized at the top of the list of overdue patients generated by Simple. Nurses call overdue patients through a toll-free, anonymized service with a single click. Automatic reminder messages are sent to patients who miss visits, progress can be monitored in real-time, and performance can be monitored daily or monthly. Reports are generated automatically, saving time spent compiling and verifying paper records.

The project was implemented in a phased manner in 26 districts across five states between January 2018 and 31st December 2019. The project districts are home to an estimated 4.5 million people with hypertension, of whom 570,365 (12.7%) were registered for treatment. Among them, 89% (510,856) were actively under care in public sector health facilities, and the remaining were lost to follow up at least through March of 2020, according to available records. Thus, 11% of all people estimated to have hypertension in the 26 districts were actively under care in IHCI facilities in the program’s first two years.

**Fig. 2:**
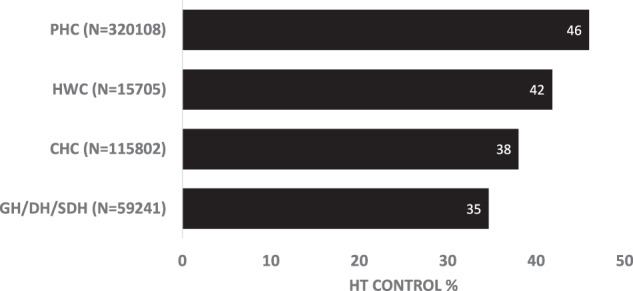
Blood pressure control in various types of facilities in Jan-March, 2020 among a cohort of patients under care in 26 districts in India.

Clinic-level BP control was 43% across all IHCI districts in the five states during the most recent visit between 1st January and 31st March 2020. BP control was highest in IHCI facilities closer to the patient’s home, such as PHC (46%) and Health and Wellness Centres (42%). District hospitals had the lowest control (35%) (Fig. 2). There was a wide variation of clinic-level BP control across districts, ranging from 22% to 79% during the most recent visit in Jan-March, 2020 (Table 2). Control was above 50% in 10 districts in Telangana (a southern state). Six districts in three states had control of 40%- 50%.

**Table 2:** Patients under the care and proportion of controlled blood pressure (BP), uncontrolled BP, and missed visits among patients on treatment in public sector facilities in 26 districts in India, 2020.

State	District	Population >30 yrs (in thousands)	Estimated Hypertensives	Patients under care	Proportion of hypertensives under care	BP control (*n*)	BP control (%)	Uncontrolled BP (*n*)	Uncontrolled BP (%)	Missed visit (*n*)	Missed visit (%)
TL^a^	Peddapally	398	76,356	8751	11	6844	78	596	7	1211	14
TL	Rajanna Siricilla	276	52,998	6144	12	4697	76	686	11	700	11
TL	Jagityal	493	94,605	9944	11	7119	72	1276	13	1436	14
TL	Warangal (Rural)	352	81,425	7637	9	5435	71	460	6	1619	21
TL	Karimanagar	503	96,554	8303	9	5566	67	946	11	1691	20
TL	Mulugu	154	35,475	3929	11	2467	63	522	13	861	22
TL	Jangoan	278	62,237	9052	15	5388	60	863	10	2692	30
TL	J Bhupallapally	217	50,173	4108	8	2299	56	326	8	1413	34
TL	Warangal (Urban)	530	122,483	9997	8	5492	55	1353	14	3027	30
TL	Mahabubabad	380	87,772	7167	8	3788	53	942	13	2384	33
MH^b^	Sindhudurg	456	103,543	17,413	17	8573	49	3911	22	4822	28
MP^c^	Chindwara	958	217,386	21,386	10	10,317	48	3140	15	7848	37
MP	Ratlam	679	133,756	10,759	8	5021	47	1321	12	4417	41
KL^d^	Kannur	1386	310,528	57,369	18	26,422	46	21,238	37	9445	16
KL	Trivandrum	1844	566,041	78,584	14	33,164	42	27,049	34	18,195	23
KL	Wayanad	421	132,191	19,984	15	8372	42	5276	26	6305	32
PB^e^	Pathankot	301	90,416	3966	4	1511	38	887	22	1540	39
MH	Satara	1564	273,647	34,018	12	12,928	38	5382	16	15,521	46
KL	Thrissur	1800	473,435	75,755	16	28,681	38	28,743	38	18,191	24
PB	Gurdaspur	819	245,655	16,808	7	6109	36	3508	21	7035	42
PB	Bathinda	731	213,461	11,171	5	4055	36	1605	14	5461	49
MH	Bhandara	595	141,512	19,472	14	5907	30	4018	21	9527	49
MH	Wardha	670	122,575	30,106	25	8474	28	6386	21	15,196	50
PB	Mansa	383	171,397	5772	3	1532	27	1239	21	2973	52
PB	Hoshiarpur	802	268,578	18,081	7	4455	25	3869	21	9693	54
MP	Bhopal	1199	317,797	16,814	5	3724	22	4191	25	8890	53
	Overall	18,188	4,541,994	512,490	11	218,340	43	129,733	25	162,093	32

^a^Telangana (TL).

^b^Maharashtra (MH).

^c^Madhya Pradesh (MP).

^d^Kerala (KL).

^e^Punjab (PB).

Uncontrolled BP among patients who visited a health facility in Jan-March, 2020 ranged from 6% to 38% (overall 25%) across districts (Table 2). Of the 26 districts, 12 had uncontrolled BP above 20%, including all four in Kerala. The proportion of patients who missed visits in the first quarter of 2020 ranged from 12% to 54% (overall 32%) in various districts (Table 2).

**Fig. 3:**
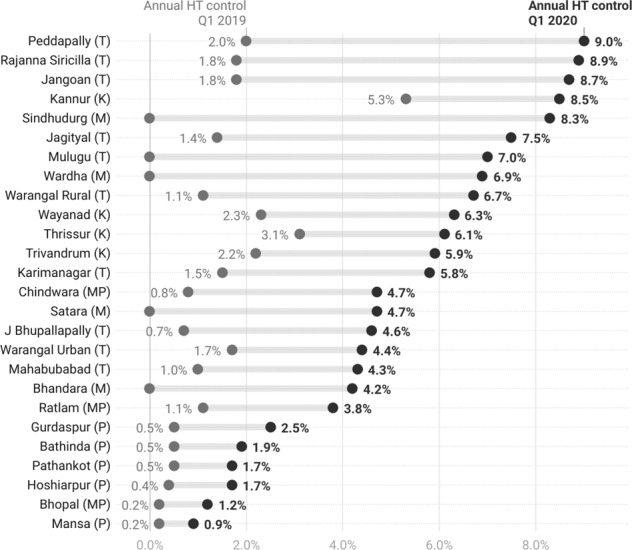
Community level blood pressure control in IHCI project districts, India. Estimated community-level BP control in 26 districts among patients under care in Jan-March, 2020 compared to Jan-March, 2019, India. (Estimated hypertensives *N* = 45,41,994, Number with BP control = 2,18,340 in Jan-March 2020, Number with BP control = 64,704 in Jan-March, 2019).

Overall estimated community-level hypertension control based on districts’ estimated number of people living with hypertension increased from 1.4% to 5.0%. We documented improvement in all project districts from Jan- to March 2020 compared to Jan-March 2019 (Fig. 3). Community-level hypertension control reached above 5% in 13 districts, including seven from Telangana, all four from Kerala, and two from Maharashtra.

## Discussion

Even in the beginning phase of the IHCI, the project demonstrated that it is possible to implement high-quality treatment regimens for hypertension with a reliable drug supply within India’s primary health care system. The project also established systematic and accurate information systems to track individual patient blood pressures and facility, district, and state-level hypertension control rates. Supportive supervision was critical for quality improvement. However, there were many challenges in implementation as NCD are still not high on the priority agenda in many states. The lack of preparedness of the health system to cater to NCD and poor awareness in the community were major barriers in the implementation of IHCI. The health system was poorly equipped to procure adequate drugs, and high-quality blood pressure monitors and human resources were inadequate to cope with NCD-related services. The lack of awareness regarding regular treatment among patients and poor linkages between clinics and community led to poor retention in care.

States selected drug- and dose-specific protocols with amlodipine as first-stage, telmisartan as second-stage, and a diuretic (chlorthalidone or hydrochlorothiazide) as the third-line drug. The WHO HEARTS package and new hypertension treatment guidelines from WHO include several sample protocols, several of which advise ARB or a fixed-dose combination as the first-line drug. [7, 13] However, in the IHCI experience, most hypertension experts across states preferred amlodipine monotherapy as the first-stage treatment due to limited access to laboratory testing in rural areas and also lower cost. One purpose of protocols was to simplify the prescription and procurement process, especially for the primary care facilities, which was critical to overcoming the drug stockouts in the project districts. Inadequate availability of antihypertensive drugs at the time of initiation of the project was consistent with a national-level survey done in 2017–18, which documented gaps in the availability of NCD drugs across public sector facilities in India. [14] Drug and dose-specific protocols, including only three drugs, enabled forecasting, simplified the procurement process, and decreased medication unit costs. This was evident because most healthcare facilities in the states had stocks for one-month refills by 2020. All three drugs were low-cost in India’s public sector facilities, and one-year treatment for a patient cost a weighted average of less than three US dollars. A study from Latin America and Caribbean countries documented the feasibility and acceptance of simple treatment protocols in the primary care settings under the HEARTS in Americas initiative. [15] Based on the experience in the initial five states, more than 15 other Indian states have developed drug- and dose-specific treatment protocols. [11]

The IHCI rigorously monitored the program’s quality by following outcomes for cohorts of patients using standard indicators of hypertension control and retention in care. World Hypertension League and WHO recommended a set of core indicators to monitor hypertension control at the community and clinic level. [7, 16] The project documented the feasibility of data collection for key indicators in primary care settings. One of the WHO HEARTS indicators, community-level BP control, estimated the overall progress in district-level blood pressure control over time and the population coverage of the program. We used clinical records to estimate the numerator and survey data to estimate the denominator to estimate the community level BP control, an indicator of program coverage and impact. The indicator suggested the progress was highly variable, and several districts made remarkable progress in one year. The limitations of the indicator are that the denominator of the number of hypertensive patients is indirectly estimated based on survey data, and the numerator for the estimation is based on only public sector facilities, although a large proportion of patients take treatment, at least episodically, in the private sector facilities.

Clinic-based indicators provide insight regarding the effectiveness of treatment in achieving control, yet only a few LMIC report these indicators routinely in the health system. One of the best examples in Thailand is a countrywide information system to track blood pressure control of all patients under care using a combination of paper-based and digital systems. In 2019, hypertension control was 59% (range: 51–67%) in public sector facilities in Thailand based on the most recent BP reading. [3] A cluster randomized controlled trial in Bangladesh, Pakistan, and Sri Lanka (*N* = 2645) documented significant improvement in BP control after implementing a multicomponent intervention in public sector clinics. [17] We documented more than 40% control in more than half of the districts. Although we initially used paper-based records, we learned that retrieval of records was complex in crowded or peripheral facilities with large numbers of patients in treatment and without an adequate workforce. Hence, the transition was made to digital records in several districts using a very easy-to-use app, which helped make the real-time monitoring of clinic-based indicators possible.

Overall, blood pressure control was higher in the primary health care facilities across all states, suggesting that decentralization to more basic, community-based facilities increases retention and control. Before the project implementation, most patients visited district hospitals or other higher-level health facilities for hypertension care. We considered the availability of drugs (in patient-days) and missed visit in the previous three months as a surrogate of the quality of care which may improve the utilisation of the healthcare facilities. Adequate availability of drugs suggests that the supply chain is functioning well. Low missed visits indicate that the health system is geared to retain patients in care by using a combination of strategies. As drug availability improved in the community-located facilities, patients could obtain refills in the primary health centres or Health and wellness centres closer to their homes. Interventions in primary care have improved hypertension control in many LMIC, including Thailand, Cuba and Peru. [3, 18, 19] The studies from Cuba and Peru were done in a small sample in a defined geographical area. [18, 19] On the other hand, Thailand implemented a nationwide program which led to 30% community level hypertension control by 2014. [3]

We documented the scalability of primary care-based hypertension management for half a million patients over two years. The unique attributes of India’s health care system which might have influenced the outcomes include procurement of generic drugs by the government procurement agencies at a very low cost, provision of free antihypertensive drugs to patients across all districts with one month refills, wide network of primary health care facilities and easy to access Health Wellness Centres with a specially trained nurse. One of the challenges we could not fully address was retention in care. We continue to work with the health facilities to assign patients to Health and Wellness Centres which are closer to home for patients. Further analysis is underway to design and evaluate strategies to increase patient registration and control. We observed a lack of card updates in a few sites using a paper-based system and incomplete data entry in the app in a few clinics. The data presented on treatment outcomes was collected from paper-based or app-based records in the health care facilities and not collected in research mode. Hence, there might be data quality issues. We tried to overcome this challenge by ensuring supportive supervision of at least 15 health facilities every month to monitor data quality. The assessment and analysis denote the real word implementation setting of a program.

We demonstrated that IHCI strategies, namely a drug- and dose-specific treatment protocol, availability of adequate protocol drugs, one-month dispensing and refills in primary care, and cohort-based monitoring of key indicators were feasible and scalable in low-resource settings in India. Based on the lessons learnt in the initial 26 districts, the project has been scaled to cover more than 100 districts across all of India’s states. Best practices were disseminated to stakeholders and will be included in the national program. To further improve treatment coverage and blood pressure control in the IHCI, we will need to strengthen the timely purchase and distribution of medications, consider provision of longer than one-month refills for controlled patients, ensure proper use of validated digital blood pressure monitors, screening of every adult attending health facilities, and maintain rigorous data quality standards based on real-time data collection using a digital software platform. Patient enrolment and follow-up should preferably be done in primary care facilities, which provide more patient-centered hypertension services and have the potential to increase patient retention in care. Research priorities include in-depth analysis to understand predictors of control, understand compliance to treatment protocols among treatment providers, and reasons for missed visits among patients.

### Summary

#### What is known about this topic

- Hypertension treatment is one of the best buys for noncommunicable disease control.
- Although hypertension can be treated with low-cost generic antihypertensive drugs in primary care settings, coverage of hypertension treatment is low.
- Only one in ten people with hypertension have blood pressure under control.

#### What this study adds

- We demonstrated a scalable hypertension control program in 26 districts enrolling over half a million patients in two years in India.
- The India Hypertension Control Initiative strategies, namely a drug- and dose-specific treatment protocol, uninterrupted supply of drugs, one-month dispensing and refills in primary care, and cohort-based monitoring of key indicators were feasible and scalable in low-resource settings.

## Data Availability

Data are available from the corresponding author at request.
